# AMPK Signaling in Energy Control, Cartilage Biology, and Osteoarthritis

**DOI:** 10.3389/fcell.2021.696602

**Published:** 2021-06-22

**Authors:** Dan Yi, Huan Yu, Ke Lu, Changshun Ruan, Changhai Ding, Liping Tong, Xiaoli Zhao, Di Chen

**Affiliations:** ^1^Faculty of Pharmaceutical Sciences, Shenzhen, China; ^2^Research Center for Human Tissues and Organs Degeneration, Shenzhen Institute of Advanced Technology, Chinese Academy of Sciences, Shenzhen, China; ^3^Clinical Research Centre, Zhujiang Hospital, Southern Medical University, Guangzhou, China; ^4^Menzies Institute for Medical Research, University of Tasmania, Hobart, TAS, Australia

**Keywords:** AMPK, energy balance, chondrocyte, osteoarthritis, signaling interaction

## Abstract

The adenosine monophosphate (AMP)–activated protein kinase (AMPK) was initially identified as an enzyme acting as an “energy sensor” in maintaining energy homeostasis via serine/threonine phosphorylation when low cellular adenosine triphosphate (ATP) level was sensed. AMPK participates in catabolic and anabolic processes at the molecular and cellular levels and is involved in appetite-regulating circuit in the hypothalamus. AMPK signaling also modulates energy metabolism in organs such as adipose tissue, brain, muscle, and heart, which are highly dependent on energy consumption via adjusting the AMP/ADP:ATP ratio. In clinics, biguanides and thiazolidinediones are prescribed to patients with metabolic disorders through activating AMPK signaling and inhibiting complex I in the mitochondria, leading to a reduction in mitochondrial respiration and elevated ATP production. The role of AMPK in mediating skeletal development and related diseases remains obscure. In this review, in addition to discuss the emerging advances of AMPK studies in energy control, we will also illustrate current discoveries of AMPK in chondrocyte homeostasis, osteoarthritis (OA) development, and the signaling interaction of AMPK with other pathways, such as mTOR (mechanistic target of rapamycin), Wnt, and NF-κB (nuclear factor κB) under OA condition.

## Introduction

Advanced technology and medical therapy in health care are highly demanded in the era after the Millennium. World Health Organization introduced the concept of “healthy aging” based on the statistics demonstrating that (1) the faster pace of the growing of aging population in the past 30 years; (2) by 2050, the proportion of the world population older than 60 years will reach nearly 22%; (3) the occurrence of chronic and metabolic diseases, such as cardiovascular disease, Alzheimer disease, diabetes mellitus, and cancer, has been dramatically increased in the upper- to middle-income and high-income countries between 2000 and 2019^1^. So, the improvements in medical care are key mission required in the future to reduce social–economic burden. Regarding to this situation, chronic metabolic diseases have been gained significant attention in the research field where scientists are aiming to increase life quality and extend life expectancy via controlling the initiation and/or the progress of chronic metabolic diseases (Aburto et al., 2020). Previous studies have indicated that there is a negative correlation between metabolic diseases and energy expenditure. It illustrated that low energy expenditure is associated with high risk of obesity (Zurlo et al., 1990; Lam and Ravussin, 2016). High energy expenditure levels and low body weight may protect people from potentially atherogenic diet (Mbalilaki et al., 2010; Ross et al., 2020).

**FIGURE 1:**
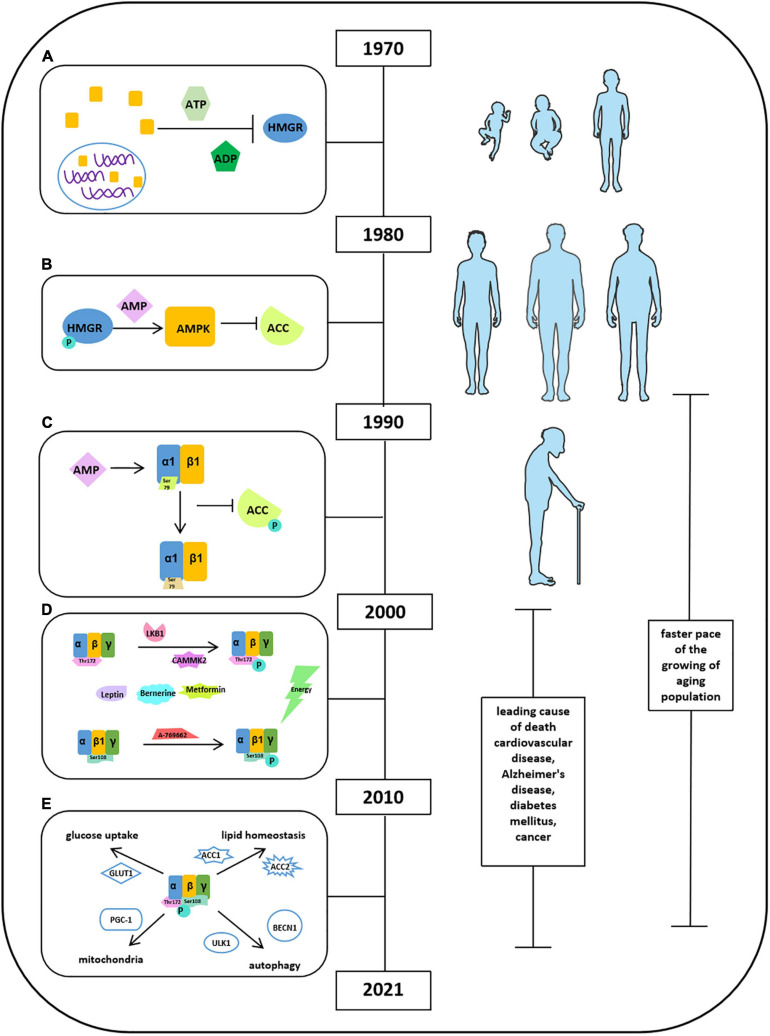
Milestones of discoveries of AMPK protein. **(A)** Cytosolic factor could inactivate 3-hydroxy-3-methyl-glutaryl-coenzyme A reductase (HMGR) in the presence of ATP and ADP. **(B)** ACC and HMGR could be purified separately, and the ACC and HMGR are regulated by AMP. **(C)** The structure of AMPK comprised an α1 catalytic subunit with a functional domain and a β1 subunit. AMPK is allosterically activated by AMP to phosphorylate and inactivate ACC at Ser79. **(D)** Factors reported to stimulate AMPK activation. **(E)** Activities of AMPK in glucose uptake, lipid homeostasis, autophagy, and mitochondria.

Brown et al. (1975) found a cytosolic factor could inactivate 3-hydroxy-3-methyl-glutaryl-coenzyme A reductase (HMGR) in the presence of adenosine triphosphate (ATP) and ADP, as shown in Figure 1A (Steinberg and Carling, 2019). Thereafter, several studies have been focused on the origin and function of this factor. They found that a self-regulated phosphorylation of HMGR kinase could form a protein kinase cascade, alone with adenosine monophosphate (AMP) activating a protein kinase but inactivating acetyl-CoA carboxylase (ACC). It has been demonstrated that ACC and HMGR could be purified separately, and the ACC and HMGR are regulated by AMP (Figure 1B). When the scientists discovered and named molecule AMPK in 1990s, Crute et al., and Bateman described the structure of AMP-activated protein kinase (AMPK) comprised an α1 catalytic subunit with a functional domain and a β1 subunit. Most importantly, it has been reported that AMPK is allosterically activated by AMP to phosphorylate and inactivate ACC at Ser79, implying the function of AMPK in an allosteric and covalent activation manner (Figure 1C). This laid the foundation for all the future studies on exploring the functional activation of AMPK. Meanwhile, a few attempts were conducted and tried to study the functions of AMPK in tissue distribution associated with metabolic physiological conditions: glycogen synthase in skeletal muscle related to GLUT4 translocation and lipolysis and lipogenesis in isolated rat adipocytes with AICAR (Steinberg and Carling, 2019). The limitation of these studies may be due to the shortage of scientific knowledge and appropriate measurement techniques. After the Millennium, with the great leap forward of science and technology, the scientists have ushered in a prosperous age that advanced technology applied to explore comprehensive molecular mechanisms in detail. In this transition period, gamma subunit of AMPK had been revealed and implied its function in mediating energy; thus, peroxisome proliferator-activated receptor δ (PPARδ) agonists were applied in few studies. In addition, besides the depth in understanding the activation and deactivation of AMP, depending on allosteric stimulation, autoinhibitory effects of AMPK also pointed out by Pang et al. To expand the understanding of signaling pathway of AMPK, several factors have been identified (Figure 1D). Liver kinase B1 (LKB1) and Ca^2+^/calmodulin-dependent protein kinase kinases (CaMKK2) are the upstream kinases phosphorylating AMPK at Thr172. Sanders et al., illustrated that A-769662 was a valuable tool for AMPK activation, and ADaM site activators activate AMPK at Ser108 at the β1 subunit. Leptin and adiponectin both stimulate fatty acid oxidation by activating AMPK. Berberine and metformin acted as antidiabetic effects via activating AMPK activities. On the basis of this, the idea of APMK-allied food intake has leaped into public attention. AMPK has been shown to be involved in glucose transport in skeletal muscle, orexigenic neuropeptide inhibition, mitochondrial biogenesis, circadian clock regulation, and promotion of autophagy. Furthermore, the idea “energy sensor” of AMPK has been pointed out as the response to the changes of intracellular AMP, ADP, and ATP. In 2010s, the structural domains of AMPK were fully characterized, including an α subunit with a catalytic domain, β-regulatory subunits, and a γ-regulatory subunit. Moreover, Xiao et al., thought the high efficiency of AMPK activator 991 and A769662 was due to their high potency in tight binding to carboxy-terminal (CBM, C-interacting helix) of the β1 subunit (Steinberg and Carling, 2019). Apart from only explaining the activity of AMPK involved in certain pathophysiological conditions, in these time periods, researchers were eager to clarify the underlying mechanism in regulation of energy homeostasis to treat metabolic diseases. Carling et al., have defined AMPK as an energy sensor through phosphorylation of both ACC and HMG-CoA reductase in response to the changes in ADP/ATP concentration. Other groups have been working on observing activities of AMPK in aforementioned conditions such as glucose uptake and lipid homeostasis and identified several novel molecules (Figure 1E). For instance, AMPK regulated glucose uptake via GLUT1, controlling lipid homeostasis via phosphorylation of ACC1 and ACC2, and downregulation of their activity in tumor cells by Akt phosphorylation. AMPK also increased autophagy and mitophagy through phosphorylation of ULK1 and BECN1 at threonine 388. Moreover, the role of AMPK in mitochondrial homeostasis was well-established as regulation of PGC-1α expression in mouse adipose tissue and skeletal muscle. Next, Price et al., found that resveratrol (an AMPK activator) functions as an antioxidant through activation of AMPK in mitochondria. In addition, salicylate, biguanides, and canagliflozin were also found as AMPK activators in controlling metabolic process including glucose transformation, fatty acid oxidation, and cancer cell proliferation. A big step at this stage was that AMPK has been shown to play a key role in interorgan interaction. Reduced AMPK-ACC and mechanistic target of rapamycin (mTOR) signaling were reported in muscle of aged male in mankind. AMPK and hypoxia-inducible factor (HIF) together regulated adipogenesis via miR-455 (Steinberg and Carling, 2019). AMPK also interacts with nuclear factor κB (NF-κB) to increase p53 activity in liver cancer cells (Steinberg and Carling, 2019). Under extensive studies of AMPK functions in the past three decades, knowledge has been gained regarding AMPK structure and functional activities and signaling pathways in certain pathological conditions; however, limited information has been gained in recent years regarding roles of cardiovascular diseases, diabetes mellitus, and cancers, etc. In addition, studies on the functional activities and metabolic balance of AMPK in other organs are also needed.

## AMPK in Energy Balance

**FIGURE 2:**
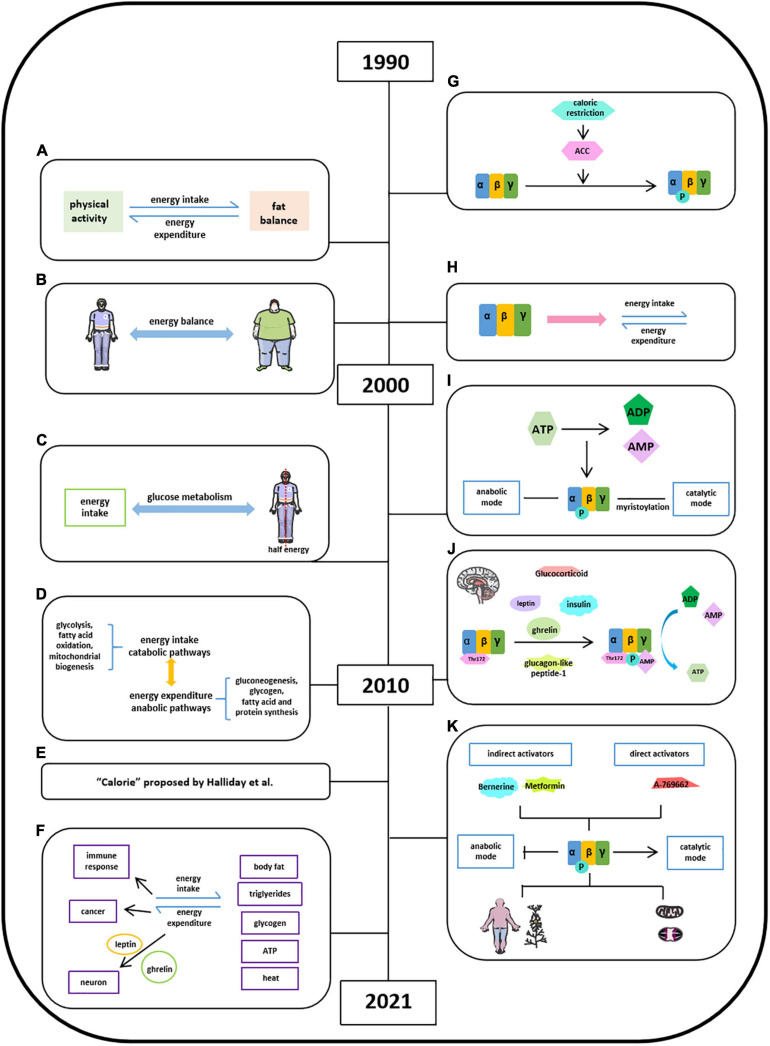
Milestones of discoveries of energy balance and the role of AMPK in energy balance. **(A)** Concept of energy balance. **(B)** Well-balanced energy is critical in maintaining a stable body weight. **(C)** Half of the body energy was obtained from glucose metabolism after energy intake. **(D)** Concept of energy intake (catabolic pathways) and energy expenditure (anabolic pathways). **(E)** The quantification of energy was measured by the unit “calorie” proposed by Halliday et al. (2011). **(F)** Format of energy store and energy balance involved activities. **(G)** AMPK is activated via inducing ACC by calorie restriction. **(H)** AMPK protein structure has been studied in energy balance. **(I)**, Energy imbalance between anabolic and catabolic process in some metabolic syndromes is due to ATP consumption. **(J)** Factors reported to stimulate AMPK activation in hypothalamus. **(K)** AMPK may act as the key regulator in energy balance in a specific organ or tissue.

Energy balance refers to the equilibrium between energy intake and energy expenditure, accompanied by energy storage, and this concept was first introduced in 1996 (Figure 2A; Hill and Commerford, 1996; Hill and Saris, 2003; Hill et al., 2013). In the following 15 years, in pursing high life quality and the encouragement of healthy diet by social media, energy balance has been linked with obesity, diet, and exercise due to a high prevalence of obesity in public epidemic worldwide (Hall et al., 2011; Hill et al., 2013; Ng et al., 2014; Manore et al., 2017). The well-balanced energy is critical in maintaining a stable body weight illustrated by several groups since 1999 (Figure 2B; van Baak, 1999; Webber, 2003; Hill et al., 2012; Hill et al., 2013; Manore et al., 2017), whereas the imbalanced energy resulted in weight loss, which could be used as the intervention of obesity (Hall et al., 2011). It is known that half of the body energy was obtained from glucose metabolism after energy intake (Figure 2C; Murphy and Bloom, 2006). When it comes to energy balance, energy intake refers to catabolic pathways (glycolysis, fatty acid oxidation, and mitochondrial biogenesis) and energy expenditure regarded as anabolic pathways (gluconeogenesis, glycogen, fatty acid, and protein synthesis) (Figure 2D; Lim et al., 2010). The quantification of energy was measured by the unit “calorie” (Figure 2E; Halliday et al., 2011). The generated energy produced by food intake could be stored as body fat, triglycerides, or glycogen in the form of ATP and dissipated as heat (Figure 2F; Hill and Saris, 2003; Halliday et al., 2011; Keith, 2013). Moyes et al. (2010) thought that there is a link between negative energy balance and impaired immune response. Gerber and Corpet (1999) proposed that prevention of visceral obesity could potentially prevent cancer via balancing caloric intake and caloric expenditure. In addition, other studies have demonstrated that energy balance is critical in neuronal activity and leptin and ghrelin control energy balance in neurons (Strassburg et al., 2008; Dietrich et al., 2010; Brown et al., 2017).

Witters et al. (1994) found AMPK is activated via inducing ACC by calorie restriction in varying nutritional states (Figure 2G; Steinberg and Carling, 2019). Hardie et al. (1999) found that AMPK monitored cellular energy changes through regulation of nucleotide concentration. Based on scattered analyses, in 2000s, with the information of AMPK protein structure, significant progress has been made in the understanding of the role of AMPK in energy balance (Figure 2H). Hardie (2004) concluded that the energy imbalance between anabolic and catabolic process in some metabolic syndromes is due to ATP consumption disorder, and several AMPK activators have promising therapeutic effects via activating AMPK phosphorylation. The AMPK isoforms α (α1, α2), β (β1, β2), and γ (γ1, γ2, and γ3) were identified in humans and rodents and were encoded by distinct genes (Figure 2I). And a year after, Steinberg and Kemp summarized a comprehensive story about the structure and regulation of AMPK in metabolism. The ratio of [ATP]/[ADP]:[AMP] was regarded to metabolic coupling of anabolic and catabolic pathways of AMPK activities known as “adenylate charge hypothesis.” Steinberg and Kemp also suggest that the metabolic stress could sense the change of AMPK levels in yeast. Moreover, the functional roles of AMPK in carbohydrate metabolism (glucose), lipid metabolism (fatty acid, mitochondrial, and cholesterol), protein synthesis, cell growth, cell apoptosis, cell polarity, and ion flux were well-established (Steinberg and Kemp, 2009). Lim et al. (2010) further empathized that the γ subunit of AMPK could tightly bind AMP in low energy states to activate AMPK with persistent phosphorylation of Thr172 residue in maintaining energy balance (Figure 2J). Leptin stimulates hypothalamosympathetic axis in an AMPK-independent manner. Adiponectin could activate and stimulate AMPK activity both *in vivo* and *in vitro*, thus leading to excitation of glucose uptake, fatty acid oxidation, and PEPCK annexation in liver and muscle. Resistin decreases fatty acid uptake and oxidation in skeletal muscle via inhibiting AMPK activity (He et al., 2018). Ghrelin and cannabinoids stimulate AMPK activity in hypothalamus and heart, while inhibiting AMPK activity in adipose tissue and liver. Insulin, as an anorectic hormone, stimulates glucose uptake and inhibits hypothalamic AMPK activity. Glucagon-like peptide-1 inhibited fasting-induced increase of hypothalamic AMPK activity as an anorectic effect. And glucocorticoids activate AMPK activity in hypothalamus via stimulating endocannabinoid synthesis (Lim et al., 2010). By 2021, accumulated evidence suggest that AMPK may act as the key regulator in energy balance in a specific organ or tissue to a certain extent (Figure 2K; Hardie, 2014; Lopez et al., 2016; Garcia and Shaw, 2017; Herzig and Shaw, 2018; Steinberg and Carling, 2019; Wang et al., 2020b).

Hardie provided the insights into stimulatory effects of AMPK on catabolic pathway and inhibitory effects of AMPK on anabolic pathways with direct targets and also listed a number of natural products, which have been reported to activate AMPK pathway and underlying mechanisms. Lopez’s group suggests that hypothalamic AMPK signaling pathway regulates energy homeostasis by integrating peripheral signals, including hormones and metabolites, with neuronal networks. Garcia and Shaw showed that AMPK, as a cellular energy sensor, restores metabolic balance, together with upstream and downstream factors. Herzig and Shaw were focusing on serine/threonine kinase AMPK complex in guarding mitochondrial homeostasis, including mitophagy and autophagy. Steinberg and Carling summarized pharmacological agents of AMPK activators, including berberine, metformin, and A769662, in the treatment of metabolic syndrome based on the information of AMPK structure and regulation. Generally speaking, the core of AMPK controls energy balance and acts as a critical bioenergy sensor, regulating anabolic and catabolic pathways under different physiological conditions, which are highly sensitive to the changes of AMP and ATP levels.

## AMPK in Chondrocytes

**FIGURE 3:**
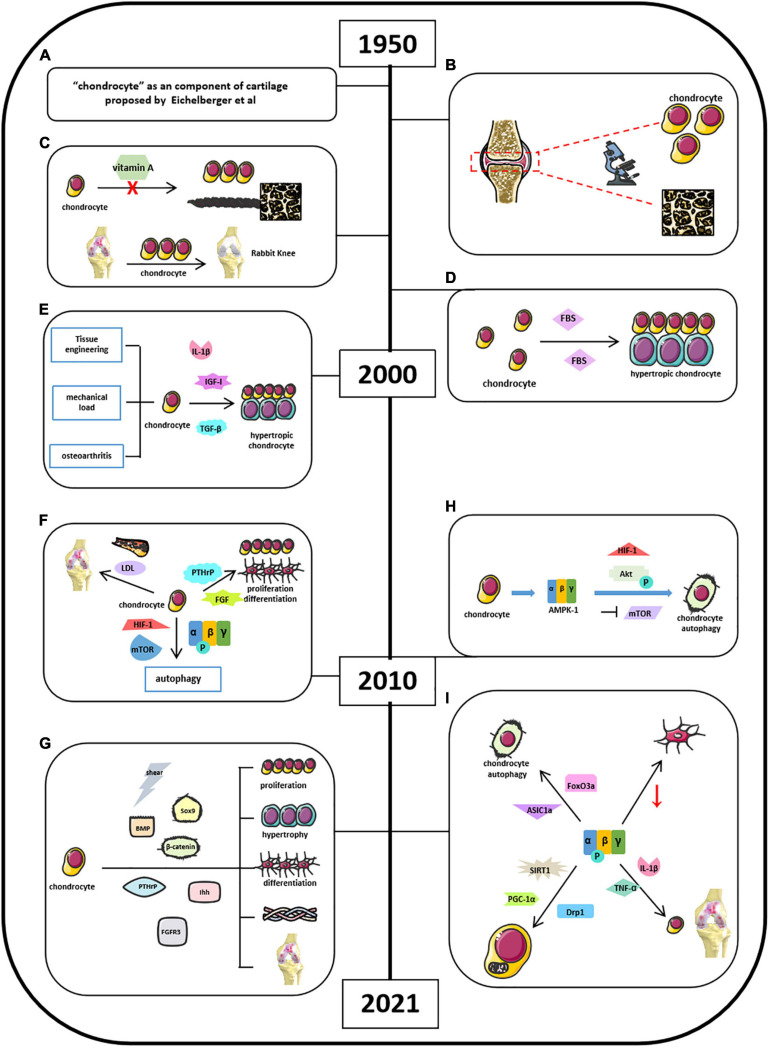
Milestones of discoveries of chondrocytes and the role of AMPK in cartilage chondrocytes. **(A)** Eichelberger et al. (1951) proposed “chondrocyte” as a component of cartilage. **(B)** Godman and Porter applied electron microscope to reveal the evolution of chondrogenesis. **(C)** Vitamin A could inhibit chondrogenesis and limiting extracellular space. **(D)** Cell density and FBS concentrations determine the transitional fate of resting chondrocytes to proliferative and hypertrophic chondrocyte. **(E)** Understanding of chondrocytes associated with osteoarthritis and upstream mediators in regulating chondrocyte activities. **(F)** Factors involved in cartilage chondrocytes’ homeostasis by 2010. **(G)** Current signaling pathways mediate chondrocyte activities. **(H)** Mouse chondrocytes expressed energy sensor AMPK-1, which promotes chondrocyte autophagy associated with Akt activation, mTOR suppression, and HIF-1 expression. **(I)** Current AMPK-related signaling pathways in mediating chondrocyte homeostasis.

Chondrocytes are derived from mesenchymal stem cells and form “hyaline cartilage” (Lee et al., 2013; Rim et al., 2020). It is well-established that chondrocytes contribute to endochondral ossification in the embryonic cartilage, thus helping bone elongation in skeletal development via self-proliferation and self-hypertrophy (Rim et al., 2020). The initial studies of “chondrocyte” could retrospect to the mid-20th century when Eichelberger et al. (1951) proposed that chondrocytes are the primary cells forming cartilage (Figure 3A). Okada (1960) found that chondrocytes had ability in generating cartilaginous matrix, and Godman and Porter applied electron microscope to reveal the evolution of chondrogenesis from chondroblasts to chondrocytes (Figure 3B; Okada, 1959; Godman and Porter, 1960). After experiencing a decade of research blank, by 1980, studies of chondrocytes back to the researchers’ scope and chondrogenesis were emerging as well. Searls’ group first showed that cartilage-forming region could be detected by light microscope (Searls et al., 1972). Vasan and Lash (1975) and Lewis et al. (1978) found that vitamin A could inhibit chondrogenesis by restraining cell proliferation, limiting extracellular space with thick and banded collagen fibrils but no proteoglycan granules (Figure 3C). Green found that chondrocytes isolated and cultured *in vitro* could repair a large articular defect when allografting took place in rabbit knee (Green, 1977). And later, in 1980s, Bruckner’s group suggests that the transition from resting chondrocytes to proliferative and hypertrophic chondrocytes depends on cell-seeded densities and fetal bovine serum (FBS) concentrations (Figure 3D; Bruckner et al., 1989). Moreover, Radomska et al. (1989) implied that chondrocytes could be used as the target in labeling cell-mediated cytotoxicity with immune organs. *In vitro* chondrocyte cultures were highly advocated in this period because of its obvious repairing capacity (Grande et al., 1989; Wakitani et al., 1989; Robinson et al., 1990). Kawamura et al. (1998) invented that the gel–chondrocyte composite could be used to treat cartilage defects better than rabbit articular cartilage. Aulthouse’s group successfully cultured human chondrocytes with ultrastructural features presented *in vitro* (Aulthouse et al., 1989). Tumor growth factor β (TGF-β) potentially modulates chondrocyte proliferation and matrix synthesis in endochondral calcification in an autocrine manner (Rosier et al., 1989; Li et al., 2010). The role of mechanical load in the development of osteoarthritis (OA) and the tissue engineering in cartilage repair are the focus of research in the last decade before the 21st century (Figure 3E; Mauck et al., 2000; Wilkins et al., 2000). Besides TGF-β, Studer et al. (2000) also indicated that chondrocytes are insensitive to anabolic actions in the presence of nitric oxide (NO), which is partially due to the inhibition of insulin-like growth factor I (IGF-I) receptor self-autophosphorylation (Figure 3E). Other studies also suggested some upstream mediators, such as interleukin 1β (IL-1β), electric fields, and thyroid hormones in the regulation of chondrocyte activities (Chao et al., 2000; Nerucci et al., 2000; Robson et al., 2000). After millennium, studies about chondrocytes were still focused on its role in bone homeostasis. Ichinose et al. (2010) well-depicted the differences among chondrocytes, bone marrow–mesenchymal stem cells (MSCs), and synovium–MSCs in cellular morphology, aggregation, and differentiation during *in vitro* chondrogenesis. Articular cartilage repair and OA remain to be a hot topic when exploring the function of chondrocytes. In addition, Dreier suggested that OA is initiated from vascularization and focal calcification of joint cartilage (Dreier, 2010). Kishimoto suggested oxidized low-density lipoprotein leads hypertrophic chondrocyte-like phenotype in OA through oxidative stress induction (Figure 3F; Kishimoto et al., 2010). Parathyroid hormone–related protein (PTHrP) and fibroblast growth factor (FGF) were found to be involved in chondrocyte proliferation and differentiation (Figure 3F; MacLean et al., 2004; Yamaoka et al., 2010). Bohensky et al. (2010) indicated that HIF-1 regulates chondrocyte autophagy via AMPK and mTOR signaling pathway (Figure 3F). By now, studies of chondrocytes were moving forward from macroregulation of cartilage integrity to the systemic mechanisms. Sharifi and Gharravi (2019) summarized a general properties of articular chondrocytes, such as matrix synthesis and its response to shear stimuli. Xing et al. (2019) indicated that osterix could mediate early postnatal growth during the formation of secondary ossification center. Bougault et al. (2014) indicated frizzled-related protein B could modulate matrix metalloproteinase induction in mouse chondrocytes via regulation of Wnt/β-catenin pathway (Figure 3G). Li and Dong (2016) conclude that bone morphogenetic protein (BMP), PTHrP, Ihh, FGFR3, Sox9, β-catenin, O_2_ tension, and reactive oxygen species (ROS) signaling pathways mediate chondrocyte formation, differentiation, maturation, and hypertrophic differentiation (Figure 3G). Other studies also mentioned the utilization of modern technique to explore the function of chondrocytes. For instance, Kim et al. (2020) found that equine bone marrow–derived cells with nanoparticles could promote the growth of chondrocytes and reduce cytokine-induced apoptosis on chondrocytes. Several other groups also illustrated that chondrocyte apoptosis was induced by NO from endoplasmic reticulum (ER) stress (Takada et al., 2011, 2013; Yamabe et al., 2013).

Chondrocyte is the only cell type in articular cartilage. AMPK signaling mainly affects chondrocyte functions during OA development, but not other cells. The roles of AMPK in chondrocyte function have been reported since 2009. It started from understanding the autophagy in chondrocytes. This might be related to the previous findings that AMPK activation was induced by ER stress leading to chondrocyte apoptosis. Srinivas et al. (2009) and Bohensky et al. (2010) found that mouse chondrocytes expressed energy sensor AMPK-1, which promotes chondrocyte autophagy associated with Akt activation, mTOR suppression, and HIF-1 expression (Figure 3H). AMPK/FoxO3a pathway was found to be involved in ASIC1α-mediated articular chondrocyte autophagy in rats (Figure 3I; Dai et al., 2017; Zhao et al., 2018; Ge et al., 2019). Bandow’s group indicated that significant decrease in phosphorylation of catalytic AMPK α subunit was found during chondrogenic differentiation of primary chondrocyte precursors (Bandow et al., 2015). Terkeltaub et al. (2011) suggest that AMPK restrained matrix degradation in chondrocytes in the presence of inflammatory cytokines IL-1β and tumor necrosis factor α (TNF-α) in both human and mouse (Figure 3I). Zheng et al. (2020) showed that activation of AMPK/Drp1/mitochondrial fission pathway mediates chondrocyte death and migration injury (Figure 3I). Moreover, AMPK participated in chondrocyte dysfunction, hypertrophy, and fibrotic differentiation (Liu N. et al., 2020; Liu Z. et al., 2020). Petursson et al. (2013) and Zhou et al. (2017) showed that downregulation of AMPK signaling resulted in inhibition of matrix catabolic responses in articular chondrocytes during OA development. Ma et al. (2018) stated that SIRT1/AMPK/PGC-1α signaling leads to mitochondrial dysfunction in chondrocytes with increased oxidative stress and apoptosis, which might be the etiology of OA (Figure 3I). Although these studies suggest that AMPK participates in the regulation of chondrocyte activity, more in-depth studies are required to further clarify the molecular mechanisms of AMPK in the regulation of chondrocyte homeostasis.

## AMPK in OA

**FIGURE 4:**
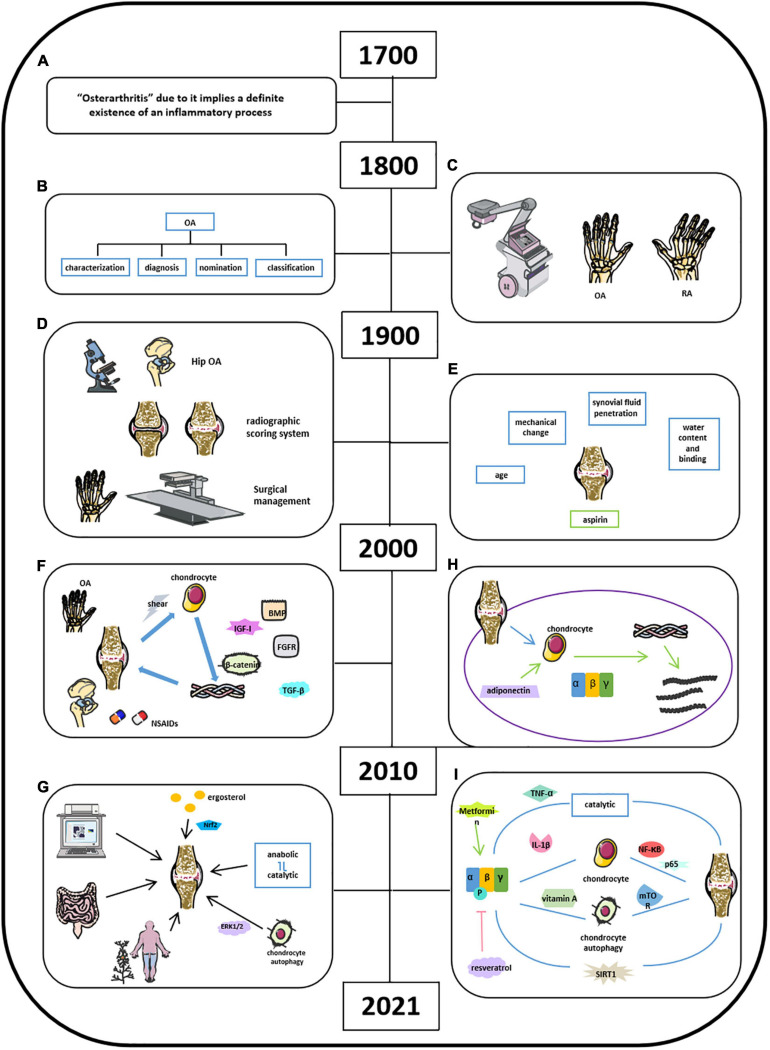
Milestones of discoveries of osteoarthritis and the role of AMPK in cartilage regeneration. **(A)** Physicians recognize the inflammatory process of OA by 18th century. **(B)** In 19th century, the knowledge of clinical OA became comprehensive. **(C)** X-rays applied in clinics helped Goldthwait distinguish OA from rheumatic arthritis (RA). **(D)** In 1900s, light microscope and electron microscope helped physicians in knee and hip OA treatment. **(E)** Knowledge of OA related in age, mechanical change, synovial fluid penetration, and water content binging. **(F)** NSAIDs and some herbal medicines were used to treat OA or relieve OA-related pain; activation of cartilage anabolic factors was associated with the degradation of proteoglycans, collagen type II, and cleaving matrix metalloproteinase. **(G)** Comprehensive understanding of OA by 2021. **(H)** Adiponectin mediates matrix degradation in human OA chondrocytes via AMPK and JNK pathways. **(I)** Molecules involved in OA homeostasis.

Osteoarthritis is characterized as a degenerative joint disease and influenced 303 million people globally, as reported in 2017 (Chen et al., 2017; Kloppenburg and Berenbaum, 2020). OA has gained great attention due to its high prevalence worldwide. Studies about OA could retrospect back to the 18th century, and physicians did not recognize the inflammatory process of OA until the 18th century (Figure 4A; Suri et al., 2012; Hardcastle et al., 2015; Peter et al., 2015; Wallace et al., 2017). Bollet suggests that the pathogenesis of OA was related to cartilage degeneration via activation of proteolytic enzyme, decrease in matrix components, and increase in penetration of synovial fluid. Our recent studies demonstrated that AMPK expression was significantly reduced in articular cartilage tissues in OA mouse model. Inflammatory cytokines were found in synovial fluid and serum in patients with RA and OA since 1970 (Figures 4B,C; Gervis, 1949; Suri et al., 2012; Hardcastle et al., 2015; Peter et al., 2015). Physicians and researchers were still trying to exploring a better way to treat OA; thus, non-steroidal anti-inflammatory drugs (NSAIDs), and some herbal medicines were used to treat OA or relieve OA-related pain, which was the major complain in clinical OA (Figures 4D,E; Long et al., 2001; Seed et al., 2009; Hu et al., 2020; Yu et al., 2021). Meanwhile, other researchers were aiming to demonstrate the potential pathobiology of OA. Besides the well-known risk factors such as aging and obesity, Abramson indicated cartilage chondrocytes as mechanosensors and osmosensors to negatively charge cartilaginous extracellular matrix in response to mechanical and osmotic stresses. And degeneration of articular cartilage leads to the activation of cartilage anabolic factors, such as BMP, IGF-I, TGF-β, and FGFs, which were associated with the degradation of both proteoglycans and collagen type II and cleaving matrix metalloproteinase (Figure 4F; Abramson and Attur, 2009). By 2021, accumulated knowledge of OA has been reported with a more broaden horizon and a more significant depth (Figure 4G). Because of the fast pace of high techniques and their application in research, studies on OA have further emphasized on molecular biology in all aspects. Chen et al. (2017) listed a series mouse model in OA studies such as traditional DMM model, aging mouse model, and available transgenic mouse models. It provided a systemic understandings of the potential pathological mechanisms of OA related to its corresponding signaling pathways, including Wnt/β-catenin, TGF-β, Ihh, FGF, and NF-κB (Zhu et al., 2009; Weng et al., 2012; Shen et al., 2013, 2014; Chang et al., 2019; Kuang et al., 2020). Moreover, Rim et al. (2020) indicated that the shifting from anabolic to catabolic signaling due to chondrocyte dedifferentiation leads to OA initiation and progression. Xu et al. (2021) found that polysaccharide from *Angelica sinensis* could attenuate OA chondrocyte apoptosis via ERK1/2 inducing autophagy. Current medicines in the treatment of OA focus on reducing pain relief and limited drugs are available in impeding OA initiation and/or progression. Traditional medicines focus on pain relief and anti-inflammation, for example, NSAIDs and vitamin D. With the building-up evidence of signaling pathways in controlling OA pathology, novel drugs targeting specific signaling molecule were developed and have shown a therapeutic potential to some extent. For examples, BMP7 limits progression of OA, FGF-18 targets cartilage of knee OA, TGF-β inhibitor targets subchondral bone remodeling, and resveratrol participates in OA chondrocyte metabolism through upregulation of SIRT1 gene (Kim et al., 2014; Zhang et al., 2019). In addition, other reports indicate that several clinical trials have been conducted to evaluate the efficacy of OA treatment drugs like fisetin, colchicine, GSK3196165, SM04690, MIV-711, Tanezumab, Fasinumab, Ampion^TM^, and so on. (US National Library of Medicine).

Although OA has been recognized as low-grade inflammatory disease with elevations in systemic inflammatory markers, such as IL-1, IL-6, IL-10, and TNF, cumulative evidence has indicated that OA is also a metabolic disorder where energy metabolic pathways, including glycolytic and TCA, are upregulated to meet the demand of the ATP for cartilage repair (Zhuo et al., 2012; Zhai, 2019). Also, diabetes could increase ROS and advanced glycosylation end products (AGEs), thus leading to further damage of articular cartilage in OA (Zhai, 2019). Accumulated studies of AMPK and OA were started to be reported since 2010. In detail, Kang et al. (2010) found that adiponectin mediates matrix degradation in human OA chondrocytes as the catabolic effect via AMPK and JNK pathways (Figure 4H). Liu-Bryan et al. (2015) summarized that inflammation disturbed cellular energy balance and increased cell stress via enhancing catabolic activities in degrading articular chondrocytes in OA (Liu-Bryan, 2015; June et al., 2016). Zhou’s group demonstrated that AMPK activity maintains joint homeostasis and OA development by enhancing IL-1β–stimulated catabolic response (Figure 4I; Zhou et al., 2017). Several groups illustrated thaactivationt of AMPK phosphorylation leads to suppressing NF-κB and its downstream molecule p65, which is involved in modulating OA cartilage in response to IL-1β–induced inflammation (Yang et al., 2019; Wang et al., 2020a; Zhu et al., 2020). Kong et al. (2020) suggest that AMPK-mTOR signaling pathway could reduce OA inflammation via activation of chondrocyte autophagy in the presence of active vitamin D. Several investigators at this time also observed the effect of metformin and resveratrol in the treatment of OA. Wei et al. (2018) showed that resveratrol suppresses induction of pAMPK and SIRT1 protein expression in OA rats. Metformin has been demonstrated to prevent cartilage degeneration and reduce pain behavior through activation of AMPK signaling (Feng et al., 2020; Li et al., 2020). Wang’s group concluded that decreased phosphorylation of AMPKα at T172 in chondrocytes aggravated a catabolic metabolism in response to inflammatory cytokines like IL-1β and TNF-α. And they also indicated that AMPK and its downstream molecule SIRT1 could switch chondrocyte metabolic activities from oxidative phosphorylation to glycolysis accompanied by increased production of inflammatory mediators and catabolic factors. Mitochondrial, autophagy, and ER stress may play a catabolic role in OA development through AMPK interacting with multiple signaling pathways (Wang et al., 2020b). Considering the presence of activation of AMPK in OA homeostasis associated with enhanced catabolic response, we suggest that AMPK could provide energy for the inflammatory reactions to OA as shown in metabolic syndrome and diabetes. Although the cumulative evidence has identified that AMPK activities are involved in OA pathogenesis, the exact and clear signaling pathways and molecular mechanisms are needed to be further investigated.

## Interaction of AMPK Signaling With Other Pathways in OA

Activated protein kinase is an evolutionarily conserved serine/threonine kinase that is vital for cellular energy metabolism homeostasis (Kahn et al., 2005). AMPK controls cellular energy status when nutrition variation sensed; once activated by low ATP status, it promotes ATP-producing catabolic pathways and shuts down ATP-consuming anabolic pathways to restore cellular energy metabolism homeostasis (Hardie, 2004). AMPK proteins are critical mediators of AMPK signaling activities and participate in extensive cross-talk with other signaling pathways. The cross-talk can occur at different levels by directly or indirectly interactions with AMPKs and three AMPK upstream kinases, which includes LKB1, TGF-β–activated kinase-1 (TAK1) and Ca^2+^/CaMKKβ. Here we discuss recent progress in our understanding of the cross-talk between AMPK signaling and signaling pathways of NF-κB, mTOR, phosphoinositide 3-kinase (PI3K)–Akt and glycogen synthesis kinase 3 (GSK3) in OA.

## NF-κB

NF-κB signaling controls inflammatory responses that develop in OA (Baker et al., 2011; Rigoglou and Papavassiliou, 2013). Zhang et al. (2020) reported that metformin protects chondrocytes against IL-1β–induced injury by regulation of the AMPK/NF-κB signaling pathway. Piao et al. (2020) showed that protectin DX attenuates IL-1β–induced OA inflammation via inhibiting AMPK/NF-κB pathway in chondrocytes and ameliorates OA progression in a rat model. However, whether AMPK directly or indirectly regulates NF-κB is unknown. Activators of AMPK, such as metformin, have been identified to have anti-inflammatory roles, and several studies have proven that AMPK inhibits NF-κB signaling through regulating distinct metabolic pathways including SIRT1 in macrophage, FOXO3 in helper T- cell activation (Lin et al., 2004) and colonic injury and inflammation (Zhou et al., 2009), PGC-1α in aortic smooth muscle and endothelial cells (Alvarez-Guardia et al., 2010), and p53 in aging mice (Salminen and Kaarniranta, 2012). Of interest, AMPK could be a potent regulator of NF-κB function in immune cells, as adiponectin was found to suppress the activation of natural killer cells and IFN-γ secretion via AMPK-mediated inhibition of NF-κB signaling (Kim et al., 2006).

## mTOR

The TOR (target of rapamycin) is a nutrient-sensing signaling pathway that is crucial for the regulation of cell growth and metabolism (Pal et al., 2015). Recent studies demonstrated that mTOR signaling plays a critical role in the development of OA. mTOR signaling pathway was suppressed by IL-1β and promotes chondrocyte autophagy and attenuates the inflammation response in rats with OA (Xue et al., 2017). Upregulation of mTOR in OA cartilage leads to increased chondrocyte apoptosis and reduced chondrocyte autophagy-related genes during OA (Zhang et al., 2015). Furthermore, miR-4262 and miR-27a have been shown to activate the PI3K/AKT/mTOR signaling pathway, indicating that targeting the mTOR signaling cascade by epigenetic regulation regulates chondrocyte viability, autophagy, and apoptosis (Sun et al., 2018; Cai et al., 2019). In addition, bioactive lipids, such as exogenous and endogenous n-3 polyunsaturated fatty acids, were reported to reduce mTORC1 and promote autophagy in chondrocytes (Huang et al., 2014). The AMPK and TOR pathways are interlinked, opposing signaling pathways involved in sensing nutrients and energy variation and regulation of cell growth (Hardie, 2014; Gonzalez et al., 2020). Previous studies have identified that activation of AMPK inhibits the mTORC1 complex by mechanisms of AMPK phosphorylating TSC2 at Thr1271 and Ser1387 (Inoki et al., 2003) and AMPK directly phosphorylating the RAPTOR component of mTORC1 at Ser722 and Ser792 (Gwinn et al., 2008). In OA, regulation of AMPK signaling pathway by mTOR may account for regulating autophagy signaling and the balance of cellular matrix metabolism in articular cartilage (Zhang et al., 2015). However, whether the above mechanism of AMPK switches off the mTOR signaling existing in chondrocytes remains to be determined.

## PI3K-AKT and GSK3

Akt, also named protein kinase B, is a serine/threonine-specific protein kinase and plays a key role in cellular metabolism, proliferation, apoptosis, and migration. Akt is activated through a phosphorylation mechanism dependent on phosphatidylinositol 3-kinase (PI3K) by extracellular factors such as estrogen, serum, and insulin. Akt might play an important role in regulating chondrocyte apoptosis or survival and might be a potential target to prevent OA. Activation of PI3K/AKT signaling in synovial cells and in chondrocytes promoted synovial cell proliferation and the expression of collagenolytic matrix metalloprotease-13 and finally accelerated the hypertrophy and degradation of chondrocytes (Huang et al., 2013, 2019). Thus, it is not surprising that there should be mechanisms by which Akt downregulates AMPK in chondrocytes. Furthermore, recent research reported that treatment with asiatic acid activated AMPK and inhibited PI3K/AKT signaling *in vitro* in ACLT-induced rat OA model (Liu Z. et al., 2020). In addition, Akt also phosphorylates and inhibits GSK3 to facilitating glycogen synthesis (Cross et al., 1995). Disruption of GSK3 function within the AMPK complex leads to higher AMPK activity and cellular catabolic activities even under anabolic conditions, indicating that GSK3 also acts as a critical sensor for anabolic signaling to inhibit AMPK (Suzuki et al., 2013).

## Conclusion

Sedentary at both work and home along with high-sugar and high-fat diet in the modern daily life caused a series of chronic metabolic diseases such as obesity and cancer. Considering the pursuit of higher life quality with longer life expectancy, public awareness of healthy lifestyle, regular diet structure, and fitness endurance are enhanced in every social aspect. OA has been observed for centuries but still has not been solved yet because of its unclear pathogenicity, although its association with age, mechanical loading, and hereditary has been explained to some extent. The clinical treatment of OA evolved from traditional surgical excision and knee joint replacement, anti-inflammation and relieving pain to blocking its initiation and progression, and focusing on prevention from intervening obesity and mechanical loading as well. Nevertheless, researchers are willing to invent therapeutic interventions to prevent or even cure OA. Hence, definitive pathological mechanisms causing OA need to be further clarified. AMPK, as it has been studied for decades, gained significant attention due to its vital role in maintaining energy balance in the body. Although its functional responsibilities in regulating skeletal developmental–related disease have not been well-depicted, its activities retaining chondrocyte balance have been well documented. Besides the well-established structure and function of AMPK at Ser79/Thr172, other allosteric activation sites of AMPK may also need to be identified and investigated. As mentioned previously, the therapeutic effects of metformin and resveratrol (AMPK activators) in the treatment of OA in mice and even in non-human primates have been demonstrated by several research groups (Wei et al., 2018; Feng et al., 2020; Li et al., 2020). Besides, metformin has been suggested as a novel drug to the patients with rheumatoid arthritis because it activates AMPK signaling, leading to the inhibition of mTOR and the differentiation of T cells *in vitro* and *in vivo*, thus reducing the invasion of fibroblast-like synovial cells (clinicaltrials.gov). These findings suggest that AMPK signaling molecules may serve as the potential drug targets for the treatment of arthritis. Although more evidence is still needed, promising results from clinical studies using AMPK-mimicking drugs with high efficacy and low toxicity have been used for OA treatment.

## Author Contributions

All authors listed have made a substantial, direct and intellectual contribution to the work, and approved it for publication.

## Conflict of Interest

The authors declare that the research was conducted in the absence of any commercial or financial relationships that could be construed as a potential conflict of interest.

## Footnotes

1 https://www.who.int
